# ANNOUNCEMENTS & RESOURCES

**Published:** 2018-02-08

## Courses

### MSc Public Health for Eye Care, London School of Hygiene & Tropical Medicine

Fully funded scholarships are available for Commonwealth country nationals. The course aims to provide eye health professionals with the public health knowledge and skills required to reduce blindness and visual disability.

For more information visit **www.lshtm.ac.uk/study/masters/mscphec.html** or email **romulo.fabunan@lsthm.ac.uk**

### Free online courses

**ICEH Open Education for eye care programme** offers a series of online courses in key topics in public health eye care. All the courses are free to access.

Courses:

Global Blindness, Eliminating Trachoma, Ophthalmic Epidemiology Basic Principles (1) and Application to Eye Disease (2).

More free courses coming! Certification also available.

For more information visit **http://iceh.lshtm.ac.uk/oer/**

## Subscriptions

Contact Anita Shah **admin@cehjournal.org**

### Subscribe to our mailing list

**web@cehjournal.org** or visit **www.cehjournal.org/subscribe**

### Visit us online

**www.cehjournal.org www.facebook.com/CEHJournal https://twitter.com/CEHJournal**

